# Nicotinamide–2,2,2-trifluoro­ethanol (2/1)

**DOI:** 10.1107/S1600536809007594

**Published:** 2009-03-11

**Authors:** Julie Bardin, Alan R. Kennedy, Li Ven Wong, Blair F. Johnston, Alastair J. Florence

**Affiliations:** a Strathclyde Institute of Pharmacy and Biomedical Sciences, The John Arbuthnott Building, University of Strathclyde, 27 Taylor Street, Glasgow G4 0NR, Scotland; b WestCHEM, Department of Pure & Applied Chemistry, University of Strathclyde, 295 Cathedral Street, Glasgow G1 1XL, Scotland

## Abstract

The nicotinamide (NA) mol­ecules of the title compound, 2C_6_H_6_N_2_O·C_2_H_3_F_3_O, form centrosymmetric *R* ^2^_2_(8) hydrogen-bonded dimers *via* N—H⋯O contacts. The asymmetric unit contains two mol­ecules of NA and one trifluoroethanol molecule disordered over two sites of equal occupancy. The packing consists of alternating layers of nicotinamide dimers and disordered 2,2,2-trifluoro­ethanol mol­ecules stacking in the *c*-axis direction. Intra­molecular C—H⋯O and inter­molecular N—H⋯N, O—H⋯N, C—H⋯N, C—H⋯O and C—H⋯F inter­actions are present.

## Related literature

For nicotinamide polymorphs, see: Wright & King (1954 ▶); Miwa *et al.* (1999 ▶); Hino *et al.* (2001 ▶). For nicotinamide co-crystals and salts, see: Fleischman *et al.* (2003 ▶); Koman *et al.* (2003 ▶); Athimoolam & Natarajan (2007*a* ▶,*b* ▶); Berry *et al.* (2008 ▶). For graph-set motifs, see: Etter (1990 ▶). For initial identification using multi-sample foil transmission X-ray powder diffraction analysis, see: Florence *et al.* (2003 ▶).
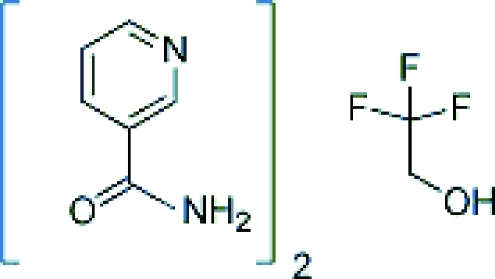


## Experimental

### 

#### Crystal data

2C_6_H_6_N_2_O·C_2_H_3_F_3_O
*M* *_r_* = 344.30
Triclinic, 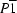

*a* = 5.0472 (3) Å
*b* = 11.2930 (7) Å
*c* = 15.0877 (10) Å
α = 107.002 (3)°
β = 96.636 (3)°
γ = 95.753 (3)°
*V* = 808.70 (9) Å^3^
*Z* = 2
Mo *K*α radiation
μ = 0.12 mm^−1^
*T* = 123 K
0.15 × 0.10 × 0.02 mm

#### Data collection

Bruker APEXII CCD diffractometer
Absorption correction: multi-scan (*SADABS*; Sheldrick, 2002 ▶) *T*_min_ = 0.903, *T*_max_ = 0.998
15936 measured reflections
4008 independent reflections
3416 reflections with *I* > 2σ(*I*)
*R*_int_ = 0.025

#### Refinement

*R*[*F* ^2^ > 2σ(*F* ^2^)] = 0.039
*wR*(*F* ^2^) = 0.114
*S* = 1.04
4008 reflections
260 parameters
H atoms treated by a mixture of independent and constrained refinement
Δρ_max_ = 0.39 e Å^−3^
Δρ_min_ = −0.29 e Å^−3^

### 

Data collection: *APEX2* (Bruker, 2007 ▶); cell refinement: *SAINT* (Bruker, 2007 ▶); data reduction: *SAINT*; program(s) used to solve structure: *SHELXS97* (Sheldrick, 2008 ▶); program(s) used to refine structure: *SHELXL97* (Sheldrick, 2008 ▶); molecular graphics: *PLATON* (Spek, 2009 ▶) and *Mercury* (Macrae *et al.*, 2006 ▶); software used to prepare material for publication: *PLATON*.

## Supplementary Material

Crystal structure: contains datablocks global, I. DOI: 10.1107/S1600536809007594/fl2234sup1.cif

Structure factors: contains datablocks I. DOI: 10.1107/S1600536809007594/fl2234Isup2.hkl

Additional supplementary materials:  crystallographic information; 3D view; checkCIF report

## Figures and Tables

**Table 1:** Hydrogen-bond geometry (Å, °)

*D*—H⋯*A*	*D*—H	H⋯*A*	*D*⋯*A*	*D*—H⋯*A*
N2—H1*N*⋯N3^i^	0.897 (16)	2.122 (16)	2.9994 (15)	165.7 (15)
N2—H2*N*⋯O1^ii^	0.898 (16)	2.013 (16)	2.9093 (13)	176.8 (14)
N4—H3*N*⋯O2^iii^	0.877 (16)	2.047 (16)	2.9139 (13)	170.1 (16)
O3—H3*O*⋯N1^iv^	0.84	1.91	2.7511 (15)	178
N4—H4*N*⋯O1^v^	0.878 (17)	2.222 (17)	3.0867 (14)	168.4 (15)
C1—H1⋯N3^i^	0.95	2.51	3.4220 (16)	161
C3—H3⋯O2^vi^	0.95	2.40	3.0103 (15)	122
C5—H5⋯F3*A*^vii^	0.95	2.46	3.408 (7)	177
C7—H7⋯O1^v^	0.95	2.36	3.2118 (14)	149
C11—H11⋯O3	0.95	2.50	3.2590 (16)	137

Symmetry codes: (i) 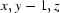; (ii) 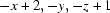; (iii) 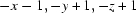; (iv) 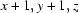; (v) 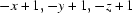; (vi) 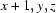; (vii) 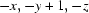.

**Fig. 1.**
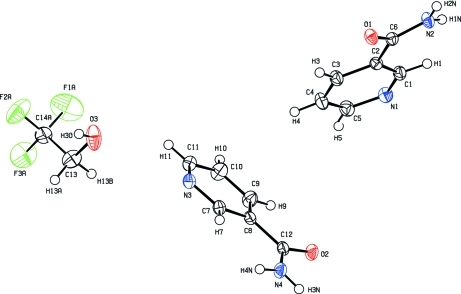
View of the title compound with the atom numbering scheme. Displacement ellipsoids for non-H atoms are drawn at the 50% probability level. One of the disordered site for the CF3 atoms has been omitted for clarity.

**Fig. 2.**
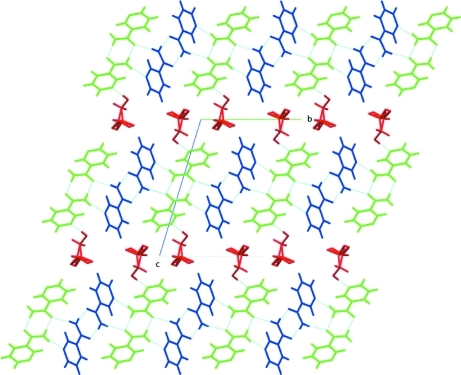
Crystal packing of the reported compound viewed down the a axis, showing the two-dimensional hydrogen-bonded network of alternating dimers, separated by layers of solvent molecules. Hydrogen bonds are shown as blue dashed lines.

## supplementary crystallographic information

### Comment

The crystal structure of nicotinamide (NA) was first reported in 1954 (Wright & King, 1954; Miwa *et al.*, 1999) and a number of polymorphic forms have also been identified (Hino *et al.*, 2001). In recent years NA has also been investigated as a pharmaceutically acceptable co-crystal former to modify the crystal structure and physico-chemical properties of drug compounds including carbamazepine (Fleischman *et al.*, 2003), salicylic acid and both racemic and S(+)-ibuprofen (Berry *et al.*, 2008). Crystal structures of 3,5-dinitrosalicylate (Koman *et al.*, 2003) as well as 2*R*,3*R*-tartrate and trifluoroacetate (Athimoolam & Natarajan, 2007*a* and Athimoolam & Natarajan, 2007*b*) salts of NA have also been reported.

In the 2,2,2-trifluoroethanol (TFE) hemisolvate reported here, the molecules crystallize in space group *P*1 with two molecules of NA and one molecule of TFE in the asymetric unit (Fig. 1). Both independent NA molecules form centro-symmetric *R*^2^_2_(8) (Etter, 1990) dimer motifs *via* N—H..O hydrogen bonds (Table 1). The anti-oriented hydrogen atoms on both amide groups form further contacts between adjacent non-symmetry equivalent dimers, either to the aromatic nitrogen, N3 (N2—H1N···N3) or the carbonyl oxygen atom, O1 (N4—H4N···O1).This gives rise to a two-dimensional hydrogen bonded layer of nicotinamide dimers lying parallel to the *a-b* plane. The remaining aromatic acceptor nitrogen atom, N1, forms an N—H···O hydrogen bond to the solvent molecule producing a structure with alternating layers of NA dimers and TFE that stack in the direction of the *c*-axis (Fig. 2). The structure is further stabilized by six weak C—H···O and C—H···F interactions (Table 1). The CF_3 _atoms of the TFE molecule are disordered over two sites with equal occupancies.

### Experimental

The novel structure reported here was discovered during a study of solvate formation in organic compounds with a range of fluorinated solvents. A crystalline sample was obtained by isothermal evaporation at 263 K from a saturated TFE solution held on a Reactarray RM2 crystalliser. Identification of the novel phase was initially made using multi-sample foil transmission X-ray powder diffraction analysis (Florence *et al.*, 2003) and a suitable single-crystal for structure determination was selected from the sample.

### Refinement

The amine H atoms were located in a difference synthesis and were refined isotropically [N—H = 0.877 (16)–0.898 (16) Å]. All other H atoms were positioned geometrically at distances of 0.95, 0.99 and 0.84 Å from the parent atoms for CH, CH_2_ and OH groups respectively. For these atoms, a riding model was used during the refinement process. The *U*_iso_(H) values were constrained to be 1.2 times *U*_eq_ of the carrier C atom or 1.5 times *U*_eq_ of the carrier O atom.

### Crystal data

**Table:**

2(C_6_H_6_N_2_O)·C_2_H_3_F_3_O	*Z* = 2
*M**_r_* = 344.30	*F*(000) = 356
Triclinic, *P*1	*D*_x_ = 1.414 Mg m^−^^3^
Hall symbol: -P 1	Mo *K*α radiation, λ = 0.71073 Å
*a* = 5.0472 (3) Å	Cell parameters from 8696 reflections
*b* = 11.2930 (7) Å	θ = 2.9–28.2°
*c* = 15.0877 (10) Å	µ = 0.12 mm^−^^1^
α = 107.002 (3)°	*T* = 123 K
β = 96.636 (3)°	Slab, colourless
γ = 95.753 (3)°	0.15 × 0.10 × 0.02 mm
*V* = 808.70 (9) Å^3^	

### Data collection

**Table:**

Bruker APEXII CCD diffractometer	4008 independent reflections
Radiation source: fine-focus sealed tube	3416 reflections with *I* > 2σ(*I*)
graphite	*R*_int_ = 0.025
φ and ω scans	θ_max_ = 28.3°, θ_min_ = 1.4°
Absorption correction: multi-scan (*SADABS*; Sheldrick, 2002)	*h* = −6→6
*T*_min_ = 0.903, *T*_max_ = 0.998	*k* = −15→14
15936 measured reflections	*l* = −20→19

### Refinement

**Table:**

Refinement on *F*^2^	Primary atom site location: structure-invariant direct methods
Least-squares matrix: full	Secondary atom site location: difference Fourier map
*R*[*F*^2^ > 2σ(*F*^2^)] = 0.039	Hydrogen site location: inferred from neighbouring sites
*wR*(*F*^2^) = 0.114	H atoms treated by a mixture of independent and constrained refinement
*S* = 1.04	*w* = 1/[σ^2^(*F*_o_^2^) + (0.0611*P*)^2^ + 0.2237*P*] where *P* = (*F*_o_^2^ + 2*F*_c_^2^)/3
4008 reflections	(Δ/σ)_max_ < 0.001
260 parameters	Δρ_max_ = 0.39 e Å^−^^3^
0 restraints	Δρ_min_ = −0.29 e Å^−^^3^

### Special details

**Table:**

Geometry. Bond distances, angles *etc*. have been calculated using the rounded fractional coordinates. All su's are estimated from the variances of the (full) variance-covariance matrix. The cell e.s.d.'s are taken into account in the estimation of distances, angles and torsion angles
Refinement. Refinement on *F*^2^ for ALL reflections except those flagged by the user for potential systematic errors. Weighted *R*-factors *wR* and all goodnesses of fit *S* are based on *F*^2^, conventional *R*-factors *R* are based on *F*, with *F* set to zero for negative *F*^2^. The observed criterion of *F*^2^ > σ(*F*^2^) is used only for calculating -*R*-factor-obs *etc*. and is not relevant to the choice of reflections for refinement. *R*-factors based on *F*^2^ are statistically about twice as large as those based on *F*, and *R*-factors based on ALL data will be even larger.

### Fractional atomic coordinates and isotropic or equivalent isotropic displacement parameters (Å^2^)

**Table:**

	*x*	*y*	*z*	*U*_iso_*/*U*_eq_	Occ. (<1)
O1	0.87250 (15)	0.11455 (7)	0.45065 (6)	0.0226 (2)	
N1	0.05569 (19)	−0.02252 (9)	0.23414 (7)	0.0240 (3)	
N2	0.7054 (2)	−0.09004 (9)	0.40991 (7)	0.0225 (3)	
C1	0.2538 (2)	−0.04184 (10)	0.29313 (8)	0.0212 (3)	
C2	0.4782 (2)	0.04629 (9)	0.33748 (7)	0.0186 (3)	
C3	0.4931 (2)	0.16131 (10)	0.32072 (8)	0.0236 (3)	
C4	0.2882 (2)	0.18307 (11)	0.26047 (9)	0.0274 (3)	
C5	0.0760 (2)	0.08876 (11)	0.21836 (8)	0.0261 (3)	
C6	0.7000 (2)	0.02482 (9)	0.40354 (7)	0.0188 (3)	
O2	−0.39941 (17)	0.44262 (7)	0.39075 (6)	0.0264 (3)	
N3	0.33815 (19)	0.66946 (9)	0.31270 (7)	0.0236 (3)	
N4	−0.2073 (2)	0.62420 (9)	0.49873 (7)	0.0236 (3)	
C7	0.1753 (2)	0.65082 (10)	0.37294 (8)	0.0219 (3)	
C8	−0.0429 (2)	0.55638 (9)	0.34852 (8)	0.0198 (3)	
C9	−0.0899 (2)	0.47587 (11)	0.25734 (9)	0.0271 (3)	
C10	0.0788 (3)	0.49317 (12)	0.19462 (9)	0.0303 (3)	
C11	0.2891 (2)	0.59119 (11)	0.22515 (8)	0.0258 (3)	
C12	−0.2296 (2)	0.53745 (10)	0.41530 (8)	0.0205 (3)	
F1A	0.5655 (16)	0.6926 (4)	−0.0538 (5)	0.0869 (18)	0.500
F1B	0.5243 (12)	0.6494 (4)	−0.0430 (5)	0.0706 (13)	0.500
F2A	0.8008 (18)	0.8692 (5)	−0.0041 (6)	0.0644 (16)	0.500
F2B	0.7971 (17)	0.8192 (6)	−0.0144 (6)	0.0647 (19)	0.500
F3A	0.4000 (15)	0.8549 (5)	−0.0620 (5)	0.0653 (16)	0.500
F3B	0.3721 (15)	0.8033 (4)	−0.0738 (4)	0.0614 (12)	0.500
O3	0.6456 (2)	0.78427 (9)	0.14649 (7)	0.0424 (3)	
C13	0.4745 (3)	0.82394 (14)	0.08562 (9)	0.0345 (4)	
C14A	0.5622 (6)	0.8123 (3)	−0.0096 (2)	0.0320 (7)*	0.500
C14B	0.5411 (6)	0.7718 (4)	−0.0096 (2)	0.0322 (7)*	0.500
H1	0.24020	−0.12000	0.30530	0.0250*	
H1N	0.592 (3)	−0.1567 (14)	0.3723 (11)	0.028 (4)*	
H2N	0.839 (3)	−0.0991 (14)	0.4511 (11)	0.030 (4)*	
H3	0.64230	0.22440	0.35030	0.0280*	
H4	0.29370	0.26120	0.24840	0.0330*	
H5	−0.06240	0.10330	0.17610	0.0310*	
H3N	−0.324 (3)	0.6138 (15)	0.5355 (11)	0.036 (4)*	
H4N	−0.094 (3)	0.6940 (15)	0.5172 (11)	0.032 (4)*	
H7	0.21110	0.70510	0.43570	0.0260*	
H9	−0.23630	0.40960	0.23810	0.0320*	
H10	0.05080	0.43890	0.13200	0.0360*	
H11	0.40360	0.60330	0.18190	0.0310*	
H3O	0.77220	0.84210	0.17440	0.0640*	
H13A	0.45750	0.91270	0.11620	0.0410*	
H13B	0.29360	0.77480	0.07530	0.0410*	

### Atomic displacement parameters (Å^2^)

**Table:**

	*U*^11^	*U*^22^	*U*^33^	*U*^12^	*U*^13^	*U*^23^
O1	0.0234 (4)	0.0156 (4)	0.0256 (4)	−0.0015 (3)	−0.0023 (3)	0.0052 (3)
N1	0.0222 (5)	0.0233 (5)	0.0252 (5)	0.0009 (4)	0.0002 (4)	0.0075 (4)
N2	0.0224 (5)	0.0162 (4)	0.0274 (5)	−0.0010 (4)	−0.0029 (4)	0.0081 (4)
C1	0.0214 (5)	0.0181 (5)	0.0244 (5)	0.0015 (4)	0.0030 (4)	0.0075 (4)
C2	0.0195 (5)	0.0167 (5)	0.0191 (5)	0.0031 (4)	0.0035 (4)	0.0044 (4)
C3	0.0243 (5)	0.0177 (5)	0.0283 (6)	0.0007 (4)	0.0023 (4)	0.0075 (4)
C4	0.0306 (6)	0.0211 (5)	0.0330 (6)	0.0033 (4)	0.0018 (5)	0.0133 (5)
C5	0.0251 (5)	0.0278 (6)	0.0274 (6)	0.0054 (4)	0.0009 (4)	0.0124 (5)
C6	0.0191 (5)	0.0167 (5)	0.0200 (5)	0.0013 (4)	0.0036 (4)	0.0051 (4)
O2	0.0278 (4)	0.0172 (4)	0.0324 (5)	−0.0040 (3)	0.0079 (3)	0.0059 (3)
N3	0.0232 (5)	0.0181 (4)	0.0300 (5)	0.0001 (4)	0.0056 (4)	0.0084 (4)
N4	0.0251 (5)	0.0189 (4)	0.0258 (5)	−0.0022 (4)	0.0061 (4)	0.0062 (4)
C7	0.0231 (5)	0.0164 (5)	0.0254 (5)	0.0003 (4)	0.0038 (4)	0.0060 (4)
C8	0.0204 (5)	0.0150 (5)	0.0252 (5)	0.0027 (4)	0.0035 (4)	0.0079 (4)
C9	0.0253 (6)	0.0224 (5)	0.0290 (6)	−0.0047 (4)	0.0035 (4)	0.0039 (4)
C10	0.0329 (6)	0.0281 (6)	0.0244 (6)	−0.0029 (5)	0.0060 (5)	0.0014 (5)
C11	0.0261 (6)	0.0246 (5)	0.0287 (6)	0.0022 (4)	0.0079 (4)	0.0103 (5)
C12	0.0202 (5)	0.0157 (5)	0.0268 (5)	0.0016 (4)	0.0029 (4)	0.0089 (4)
F1A	0.112 (4)	0.063 (3)	0.058 (2)	0.019 (3)	0.003 (2)	−0.022 (3)
F1B	0.0614 (18)	0.055 (3)	0.064 (2)	0.0127 (19)	−0.0039 (15)	−0.026 (2)
F2A	0.0451 (16)	0.110 (4)	0.048 (2)	0.003 (3)	0.0128 (15)	0.040 (3)
F2B	0.0340 (15)	0.116 (5)	0.052 (2)	0.012 (3)	0.0162 (13)	0.034 (3)
F3A	0.054 (2)	0.107 (4)	0.042 (2)	0.018 (3)	−0.0097 (16)	0.038 (3)
F3B	0.0508 (17)	0.097 (3)	0.0316 (14)	0.008 (3)	−0.0059 (11)	0.018 (2)
O3	0.0431 (6)	0.0369 (5)	0.0434 (6)	−0.0155 (4)	−0.0138 (4)	0.0212 (4)
C13	0.0278 (6)	0.0432 (7)	0.0291 (6)	0.0010 (5)	0.0013 (5)	0.0083 (5)

### Geometric parameters (Å, °)

**Table:**

F1A—C14A	1.324 (7)	C2—C3	1.3906 (16)
F1B—C14B	1.316 (7)	C2—C6	1.4978 (14)
F2A—C14A	1.289 (9)	C3—C4	1.3858 (16)
F2B—C14B	1.366 (9)	C4—C5	1.3796 (17)
F3A—C14A	1.296 (8)	C1—H1	0.9500
F3B—C14B	1.365 (7)	C3—H3	0.9500
O1—C6	1.2446 (13)	C4—H4	0.9500
O2—C12	1.2366 (14)	C5—H5	0.9500
O3—C13	1.3874 (18)	C7—C8	1.3882 (15)
O3—H3O	0.8400	C8—C12	1.5013 (15)
N1—C5	1.3421 (17)	C8—C9	1.3876 (17)
N1—C1	1.3383 (15)	C9—C10	1.3855 (18)
N2—C6	1.3311 (15)	C10—C11	1.3847 (19)
N2—H2N	0.898 (16)	C7—H7	0.9500
N2—H1N	0.897 (16)	C9—H9	0.9500
N3—C7	1.3401 (15)	C10—H10	0.9500
N3—C11	1.3361 (15)	C11—H11	0.9500
N4—C12	1.3345 (15)	C13—C14B	1.477 (3)
N4—H3N	0.877 (16)	C13—C14A	1.525 (3)
N4—H4N	0.878 (17)	C13—H13A	0.9900
C1—C2	1.3894 (15)	C13—H13B	0.9900
			
F1A···O3	2.859 (7)	C4···O2^ii^	3.1510 (15)
F1A···C10^i^	3.342 (7)	C5···C3^vi^	3.5363 (15)
F1A···F2A	2.096 (9)	C6···N1^ii^	3.2396 (14)
F1A···F3A	2.119 (9)	C6···C1^ii^	3.4524 (15)
F1B···F3B	2.118 (8)	C7···O2^ii^	3.3847 (14)
F1B···O3	2.780 (7)	C7···C12^ii^	3.4460 (15)
F2A···F3A	2.080 (12)	C7···O1^v^	3.2118 (14)
F2A···O3	2.869 (8)	C9···C11^vi^	3.5486 (15)
F2B···O3	2.750 (8)	C10···F1A^i^	3.342 (7)
F2B···F3B	2.196 (11)	C11···C9^ii^	3.5486 (15)
F3A···F1A	2.119 (9)	C11···O3	3.2590 (16)
F3A···F2A	2.080 (12)	C12···C7^vi^	3.4460 (15)
F3B···F2B	2.196 (11)	C12···C12^xii^	3.5927 (16)
F3B···F1B	2.118 (8)	C12···N3^vi^	3.2557 (15)
F1A···H10^i^	2.7300	C12···N4^xii^	3.3776 (15)
F1B···H10^i^	2.7800	C13···C1^xi^	3.4220 (18)
F2A···H3O	2.8200	C13···N1^viii^	3.4382 (18)
F2B···H13B^ii^	2.8700	C1···H13A^x^	2.9000
F2B···H3O	2.8000	C1···H1N	2.627 (16)
F3A···H5^iii^	2.4600	C1···H3O^ix^	2.8000
F3B···H5^iii^	2.5800	C3···H3N^xii^	3.086 (16)
F3B···H9^iii^	2.8600	C5···H3O^ix^	2.8900
O1···N2^iv^	2.9093 (13)	C6···H2N^iv^	2.878 (16)
O1···N4^v^	3.0867 (14)	C7···H4N	2.649 (16)
O1···C7^v^	3.2118 (14)	C7···H1^xi^	3.0500
O2···C7^vi^	3.3847 (14)	C7···H1N^xi^	2.872 (16)
O2···C3^vi^	3.0103 (15)	C12···H3N^vii^	2.984 (16)
O2···N4^vii^	2.9139 (13)	H1···N3^x^	2.5100
O2···C4^vi^	3.1510 (15)	H1···C7^x^	3.0500
O3···N1^viii^	2.7511 (15)	H1···N2	2.6100
O3···F1A	2.859 (7)	H1···H1N	2.0800
O3···F2A	2.869 (8)	H1N···N3^x^	2.122 (16)
O3···C11	3.2590 (16)	H1N···C1	2.627 (16)
O3···F2B	2.750 (8)	H1N···H1	2.0800
O3···F1B	2.780 (7)	H1N···C7^x^	2.872 (16)
O1···H7^v^	2.3600	H2N···H2N^iv^	2.57 (2)
O1···H2N^iv^	2.013 (16)	H2N···O1^iv^	2.013 (16)
O1···H4N^v^	2.222 (17)	H2N···C6^iv^	2.878 (16)
O1···H3	2.4800	H3···O1	2.4800
O2···H3N^vii^	2.047 (16)	H3···O2^ii^	2.4000
O2···H4^vi^	2.6900	H3N···C12^vii^	2.984 (16)
O2···H3^vi^	2.4000	H3N···C3^xii^	3.086 (16)
O2···H9	2.4700	H3N···O2^vii^	2.047 (16)
O3···H11	2.5000	H3O···C1^viii^	2.8000
N1···C6^vi^	3.2396 (14)	H3O···C5^viii^	2.8900
N1···C13^ix^	3.4382 (18)	H3O···N1^viii^	1.9100
N1···O3^ix^	2.7510 (15)	H3O···F2A	2.8200
N2···O1^iv^	2.9093 (13)	H3O···F2B	2.8000
N2···N3^x^	2.9994 (15)	H4···O2^ii^	2.6900
N3···C1^xi^	3.4220 (16)	H4N···O1^v^	2.222 (17)
N3···C12^ii^	3.2557 (15)	H4N···C7	2.649 (16)
N3···N2^xi^	2.9994 (15)	H4N···H7	2.0900
N4···C12^xii^	3.3776 (15)	H5···F3B^iii^	2.5800
N4···O1^v^	3.0867 (14)	H5···F3A^iii^	2.4600
N4···O2^vii^	2.9139 (13)	H7···N4	2.6000
N1···H13A^x^	2.8600	H7···H4N	2.0900
N1···H3O^ix^	1.9100	H7···O1^v^	2.3600
N2···H1	2.6100	H9···O2	2.4700
N3···H1N^xi^	2.122 (16)	H9···F3B^iii^	2.8600
N3···H1^xi^	2.5100	H10···F1A^i^	2.7300
N4···H7	2.6000	H10···F1B^i^	2.7800
C1···N3^x^	3.4220 (16)	H11···O3	2.5000
C1···C13^x^	3.4220 (18)	H13A···N1^xi^	2.8600
C1···C6^vi^	3.4524 (15)	H13A···C1^xi^	2.9000
C3···O2^ii^	3.0103 (15)	H13B···F2B^vi^	2.8700
C3···C5^ii^	3.5363 (15)		
			
C13—O3—H3O	109.00	C9—C10—C11	118.64 (12)
C1—N1—C5	117.62 (10)	N3—C11—C10	122.95 (11)
C6—N2—H2N	116.2 (11)	N4—C12—C8	118.69 (10)
C6—N2—H1N	122.9 (10)	O2—C12—N4	122.47 (10)
H1N—N2—H2N	120.7 (15)	O2—C12—C8	118.84 (10)
C7—N3—C11	117.80 (10)	C8—C7—H7	118.00
H3N—N4—H4N	117.7 (15)	N3—C7—H7	118.00
C12—N4—H4N	124.6 (10)	C8—C9—H9	120.00
C12—N4—H3N	117.4 (11)	C10—C9—H9	120.00
N1—C1—C2	123.48 (11)	C9—C10—H10	121.00
C1—C2—C6	123.54 (10)	C11—C10—H10	121.00
C3—C2—C6	118.67 (9)	C10—C11—H11	119.00
C1—C2—C3	117.78 (10)	N3—C11—H11	119.00
C2—C3—C4	119.37 (10)	O3—C13—C14B	107.67 (18)
C3—C4—C5	118.54 (12)	O3—C13—C14A	115.28 (16)
N1—C5—C4	123.19 (11)	F1A—C14A—F3A	108.0 (5)
O1—C6—C2	119.14 (9)	F1A—C14A—F2A	106.7 (5)
N2—C6—C2	118.66 (9)	F1A—C14A—C13	108.7 (4)
O1—C6—N2	122.20 (10)	F2A—C14A—F3A	107.2 (5)
C2—C1—H1	118.00	F2A—C14A—C13	113.6 (4)
N1—C1—H1	118.00	F3A—C14A—C13	112.5 (4)
C2—C3—H3	120.00	F1B—C14B—F2B	106.3 (5)
C4—C3—H3	120.00	F1B—C14B—F3B	104.3 (4)
C3—C4—H4	121.00	F1B—C14B—C13	117.2 (4)
C5—C4—H4	121.00	F2B—C14B—F3B	107.0 (5)
N1—C5—H5	118.00	F2B—C14B—C13	110.6 (4)
C4—C5—H5	118.00	F3B—C14B—C13	110.7 (4)
N3—C7—C8	123.44 (11)	O3—C13—H13A	108.00
C9—C8—C12	118.67 (10)	O3—C13—H13B	108.00
C7—C8—C9	117.85 (10)	C14A—C13—H13A	108.00
C7—C8—C12	123.48 (10)	C14B—C13—H13B	98.00
C8—C9—C10	119.30 (11)		
			
C5—N1—C1—C2	−0.71 (16)	N3—C7—C8—C9	1.42 (17)
C1—N1—C5—C4	−0.62 (17)	C9—C8—C12—N4	−171.07 (11)
C11—N3—C7—C8	−1.22 (17)	C7—C8—C12—N4	8.74 (16)
C7—N3—C11—C10	0.22 (18)	C9—C8—C12—O2	8.43 (16)
N1—C1—C2—C6	180.00 (13)	C7—C8—C12—O2	−171.77 (11)
N1—C1—C2—C3	1.37 (16)	C7—C8—C9—C10	−0.60 (17)
C1—C2—C6—O1	−169.45 (10)	C12—C8—C9—C10	179.21 (11)
C3—C2—C6—N2	−170.96 (10)	C8—C9—C10—C11	−0.30 (19)
C3—C2—C6—O1	9.14 (15)	C9—C10—C11—N3	0.5 (2)
C1—C2—C6—N2	10.45 (15)	O3—C13—C14B—F1B	58.1 (4)
C6—C2—C3—C4	−179.39 (10)	O3—C13—C14B—F2B	−64.0 (5)
C1—C2—C3—C4	−0.72 (16)	O3—C13—C14B—F3B	177.6 (3)
C2—C3—C4—C5	−0.49 (17)	O3—C13—C14A—F1A	62.9 (4)
C3—C4—C5—N1	1.21 (18)	O3—C13—C14A—F2A	−55.7 (4)
N3—C7—C8—C12	−178.39 (11)	O3—C13—C14A—F3A	−177.7 (4)

Symmetry codes: (i) −*x*+1, −*y*+1, −*z*; (ii) *x*+1, *y*, *z*; (iii) −*x*, −*y*+1, −*z*; (iv) −*x*+2, −*y*, −*z*+1; (v) −*x*+1, −*y*+1, −*z*+1; (vi) *x*−1, *y*, *z*; (vii) −*x*−1, −*y*+1, −*z*+1; (viii) *x*+1, *y*+1, *z*; (ix) *x*−1, *y*−1, *z*; (x) *x*, *y*−1, *z*; (xi) *x*, *y*+1, *z*; (xii) −*x*, −*y*+1, −*z*+1.

### Hydrogen-bond geometry (Å, °)

**Table:**

*D*—H···*A*	*D*—H	H···*A*	*D*···*A*	*D*—H···*A*
N2—H1N···N3^x^	0.897 (16)	2.122 (16)	2.9994 (15)	165.7 (15)
N2—H2N···O1^iv^	0.898 (16)	2.013 (16)	2.9093 (13)	176.8 (14)
N4—H3N···O2^vii^	0.877 (16)	2.047 (16)	2.9139 (13)	170.1 (16)
O3—H3O···N1^viii^	0.84	1.91	2.7511 (15)	178
N4—H4N···O1^v^	0.878 (17)	2.222 (17)	3.0867 (14)	168.4 (15)
C1—H1···N3^x^	0.95	2.51	3.4220 (16)	161
C3—H3···O2^ii^	0.95	2.40	3.0103 (15)	122
C5—H5···F3A^iii^	0.95	2.46	3.408 (7)	177
C7—H7···O1^v^	0.95	2.36	3.2118 (14)	149
C11—H11···O3	0.95	2.50	3.2590 (16)	137

Symmetry codes: (x) *x*, *y*−1, *z*; (iv) −*x*+2, −*y*, −*z*+1; (vii) −*x*−1, −*y*+1, −*z*+1; (viii) *x*+1, *y*+1, *z*; (v) −*x*+1, −*y*+1, −*z*+1; (ii) *x*+1, *y*, *z*; (iii) −*x*, −*y*+1, −*z*.
